# Diarrhea is a Major killer of Children with Severe Acute Malnutrition Admitted to Inpatient Set-up in Lusaka, Zambia

**DOI:** 10.1186/1475-2891-10-110

**Published:** 2011-10-11

**Authors:** Abel H Irena, Mwate Mwambazi, Veronica Mulenga

**Affiliations:** 1 Valid International, Oxford, UK; 2 Department of Pediatrics, School of Medicine, University of Zambia, Lusaka, Zambia

**Keywords:** diarrhea, HIV/AIDS, Severe Acute Malnutrition, Zambia, inpatient

## Abstract

**Introduction:**

Mortality of children with Severe Acute Malnutrition (SAM) in inpatient set-ups in sub-Saharan Africa still remains unacceptably high. We investigated the prevalence and effect of diarrhea and HIV infection on inpatient treatment outcome of children with complicated SAM receiving treatment in inpatient units.

**Method:**

A cohort of 430 children aged 6-59 months old with complicated SAM admitted to Zambia University Teaching Hospital's stabilization centre from August to December 2009 were followed. Data on nutritional status, socio-demographic factors, and admission medical conditions were collected up on enrollment. T-test and chi-square tests were used to compare difference in mean or percentage values. Logistic regression was used to assess risk of mortality by admission characteristics.

**Results:**

Majority, 55.3% (238/430) were boys. The median age of the cohort was 17 months (inter-quartile range, IQR 12-22). Among the children, 68.9% (295/428) had edema at admission. The majority of the children, 67.3% (261/388), presented with diarrhea; 38.9% (162/420) tested HIV positive; and 40.5% (174/430) of the children died. The median Length of stay of the cohort was 9 days (IQR, 5-14 days); 30.6% (53/173) of the death occurred within 48 hours of admission. Children with diarrhea on admission had two and half times higher odds of mortality than those without diarrhea; Adjusted OR = 2.5 (95% CI 1.50-4.09, P < 0.001). The odds of mortality for children with HIV infection was higher than children without HIV infection; Adjusted OR = 1.6 (95% CI 0.99-2.48 P = 0.5).

**Conclusion:**

Diarrhea is a major cause of complication in children with severe acute malnutrition. Under the current standard management approach, diarrhea in children with SAM was found to increase their odds of death substantially irrespective of other factors.

## Introduction

An estimated 8.8 million under five child deaths occurred worldwide in 2008[1]. Although the proportionate contribution of undernutrition was not established in the paper by Black RE, et al. (2010)[1], previous studies by the same author and others indicated 35% or higher percentage of under-five deaths to be attributable to undernutrition[2-4]. Sever Acute Malnutrition (SAM) affects about 20 million children globally and contributes to an estimated one million child deaths every year[5]. Over the last decade, major improvement in the survival of children with SAM treated in outpatient set-ups have been achieved [5,6]. However, the mortality rate of children with complicated SAM that receive treatment in inpatient set ups has remained unacceptably high [7]. Such high mortality in inpatient units has been attributed to either co-morbidities such as HIV infection[8] or to poor adherence to the WHO therapeutic guidelines[9].

The expansion in the coverage of outpatient treatment services is reducing the need for inpatient treatment of children with SAM. However, there will arguably be certain proportion of children with SAM that will be identified at a late stage requiring inpatient treatment to stabilize their condition. The treatment success in such inpatient set-ups is variable. It is almost impossible to stipulate with certainty the key reasons behind the successes in those institutions with low mortality or failures in others. Two underlying factors, HIV/AIDS and diarrhea infections have been documented to substantially increase the mortality rate of children with SAM receiving treatment in inpatient units[8].

The association of diarrhea and SAM is a well documented fact [10-13]. However, to date there is limited understanding of the most effective way to manage children presenting with complicated SAM and diarrhea [8]. Management is even made worse by HIV/AIDS co-morbidities [14]. HIV/AIDS infection is known to decrease the survival of children with SAM [15,16]. The degree to which diarrhea in children with complicated SAM increased their risk of mortality has also not been fully alluded to. This void is exposing children to succumb to death due to largely preventable illnesses. We analyzed data of children admitted to the Zambia University Teaching Hospital's inpatient unit to identify the prevalence of diarrhea and HIV infection and assess their effect on treatment outcome.

## Methods

### Study setting

Zambia University Teaching Hospital (UTH) is located in Lusaka, a capital city of Zambia. At the time of this study it provided the only inpatient unit for children with complicated SAM in Lusaka district. As such children receiving service in the unit come from all corners of Lusaka district. The unit has a 59 bed capacity. However, due to the large number of children needing inpatient treatment, year round, the unit has more children than it can accommodate. This is forcing cot sharing.

According to the inpatient unit audit, close to 2,000 children with SAM receive treatment in the inpatient unit annually. This constitutes close to 30% of the children with SAM that annually receive treatment in Lusaka; outpatient and inpatient combined (personal experience).

The mortality rate of SAM children admitted to the inpatient unit is over 30% (ward audit). This is despite efforts since 2001 to reduce mortality in the unit through training of staff in inpatient management of SAM as per the 1999 WHO guideline[17].

### Study population

All children 6-59 months of age admitted to the inpatient unit were eligible for the study. Children were admitted to the ward based on the presence of bilateral pitting edema and/or weight for height Z-scores (WHZ) < -3 standard deviations (SD). Weight for height Z-scores were calculated using NCHS/WHO normalized charts.

### Study design and period

This was a cohort study involving children 6-59 months old with SAM admitted to the UTH inpatient unit. The study was conducted from 1^st ^August to 31^st ^December 2009. Part of the study period (October to December) fall within the malnutrition period; December being the peak month for SAM in Lusaka.

### Sample size

Out of a total of 1041 admission that occurred between August and December 2009, 430 children between 6 and 59 months old were enrolled into the study. Children were enrolled into the study up on consent of their caregivers. Children admitted over the weekend were missed as study protocol required enrolling children within 24 hours of admission.

### Data collection

Trained ward attendants measured the nutritional status of the children. Height was measured using a stadiometer, and weight was measured to the nearest 100 g using a UNISCALE. Social and demographic data were collected using structured questionnaires. HIV serology was done using the Determine^® ^HIV-1/2 test. DNA PCR (for children under 18 months old with a positive HIV serology) was done after parental consent was obtained. MUAC was not measured as it was not part of the inpatient protocol.

On admission, all children were examined by the attending physicians. Clinical evaluation was done to assess co-morbidities. Fever was defined as an admission axilliary temperature of greater than 37.5°C. Diarrhea was diagnosed based on caregiver assessment or three or more loose stools a day.

### Clinical and nutritional care

Children were managed by a team of physicians comprising of three rotating resident physicians (average stay in the ward of 4 months) and two junior resident medical officers, supervised by one senior registrar and one consultant pediatrician. In addition, three to five nurses attended to the children in the ward.

Children were managed using WHO standard guidelines for the management of severe malnutrition. Oral vitamin A (200,000 IU if ≥ 12 months old or 100,000IU if < 1 year old) was given on admission; those with clinical signs of vitamin A deficiency received further doses on days two and 14. Children with diarrhea were given ReSoMal. A nasogastric tube was inserted into children who were assessed to be too sick to feed voluntarily or who had persistent vomiting. Children received 10% dextrose upon admission. Intravenous fluids (often 1/2 strength Darrow's solution) were used for management of shock or in children with persistent diarrhea with dehydration.

F75 therapeutic milk was used in the first phase of treatment. F75 prepared in the ward using fermented milk was given to children who continued to have diarrhea after admission. During the second phase of treatment, children were treated either with ready-to-use therapeutic food (RUTF) or F100 therapeutic milk depending on appetite test result.

Children exited from the unit on one of the following criterion; "Stabilized" if they were able to consume RUTF and were referred to one of the 25 outpatient therapeutic programs (OTP) for full recovery; "Absconders" if they were absent from the unit for two consecutive days; "Deaths" if they died while in the unit; "Transfer to AO5" if the child had tuberculosis or measles and was referred to the isolation ward.

### Outpatient treatment service

At the time of this study, outpatient service for the management of children with uncomplicated SAM was available in 25 health centre in Lusaka. Children admitted to the inpatient unit were discharged into these centers upon stabilization of their condition and were able to consume RUTF.

### Data analysis

Variables in the dataset included binary (sex, HIV, fever, WHZ score < -3SD, diarrhea, and outcome) and categorical data (nutritional status, and admission edema). Weight, height, and age were numeric data but were grouped as categorical data for purposes of analysis. During the analysis, a variable called "nutstat" was created based on a combination of children's admission edema and WHZ. Accordingly, children were classified as "Marasmic" if they had WHZ less than -3 SD but not edema, or "Kwashiorkor" if they had edema but their WHZ was ≥-3 SD, or "Marasmic-Kwashiorkor" if they had both edema and WHZ < -3 SD.

Binary outcome variable (Alive or Dead) was created. Exposure factors used included age, sex, HIV status, nutritional status, diarrhea on admission, and fever. Baseline data were compared between the two groups using mean with Standard Deviation (SD) or percentage. T-test and chi-square test was used to compare difference in mean and percentage, respectively. Variables that had a P-value of < 0.2 were modeled using logistic regression. Univariate and multivariate analysis were done by adjusting for sex, HIV, WHZ score, nutritional status, and age group. Likelihood ratio test and associated P-values were used to test association. Kaplan-Meier curves were used to estimate survival probability. Adjusted and unadjusted odds ratio, 95% confidence interval, and P-values were calculated and reported. Analysis was done using STATA 11.

### Ethical issue

Permission to conduct the study was provided by UTH, and ethical clearance was granted by University of Zambia Biomedical Research Ethics Committee. As part of the provider initiated counseling and HIV testing service offered by the hospital to all admitted patients, HIV counseling and testing was done by trained health personnel up on consent of the caretakers of children.

## Results

The majority, 55.3% (238/430) of the admitted children were boys. Almost a quarter of the enrolled children (99/430) were 6 to 12 months old. The median (IQR) age of the cohort was 17 (12-22) months. There was no significant difference in age between boys and girls (P = 0.8).

Over half of the children, 69.9% (292/418), had edematous form of malnutrition at admission, whereas, 57.0% (240/421) of the children had WHZ < -3SD. Admission weight of the children ranged from 3.2 kg to 15.5 kg, with a median admission weight of 6.5 kg (IQR, 5.5 -7.9). The boys were heavier than the girls, with a mean (SD) of 7.0 (1.7) kg compared with a mean (SD) of 6.6 (2.0) kg on admission (P = 0.02).

Of those children for whom data regarding diarrhea was present, 67.1% (255/380) had diarrhea on admission. In addition, 48.0% (182/379) reported fever on admission. HIV test results were available for 97.0% (417/430) of the children. Accordingly, HIV prevalence based on Determine^® ^HIV-1/2 tests was 38.6% (161/417) for the entire cohort and 40.6% (80/197) for those above 18 months old. Majority, 53.7% (231/430), of the children were discharged as stabilized, 40.5% (174/430) died, and 4.4% (19/430) absconded. Six children were referred to the isolation ward because they were diagnosed with tuberculosis.

The median Length Of Stay (LOS) of the cohort was 9 days (IQR, 5-14 days). The LOS for stabilized children was 10 days (IQR, 7-15). Mean LOS of children with diarrhea, 9.6 (SD, 8.1) days, was shorted than children without diarrhea, 11.8 (SD, 9.5) days, P = 0.02. LOS of children who died was 5 days (IQR, 2-10). Of the children who died, 30.6% (53/173) died within 48 hours of admission, and 65.3% (113/173) died within 1 week of admission. HIV-positive children stayed a mean (SD) of 11.9 (9.4) days, longer than HIV-negative children, who stayed a mean (SD) of 9.4 (7.6) days (P = 0.004.

Table 1 shows uni and multivariate logistic regression result. Sex, age, and admission fever had no effect on survival (adjusted P > 0.2). HIV infection was independently associated with mortality after adjusting for nutritional status and diarrhea on admission; adjusted OR = 1.6 (95% CI 0.99-2.48 P = 0.5). Those with diarrhea on admission had a two and half times the odds of death, adjusted OR = 2.5 (95% CI 1.50-4.09, P < 0.001).

**Table 1:** Univariate and multivariate analysis of factors associated with death

Variable	Outcome (died) n, (row %)	Unadjusted OR (95% CI)		P-value	Adjusted OR (95% CI)		P-value
Sex (n = 430)							
F (n = 192)	81 (46.6)	1			1		
M (n = 238)	93 (53.5)	1.14	(0.77-1.68)	0.01	1.03	(0.65-1.64)	0.8

Age group, months (n = 430)							
6-11 (n = 99	47 (27.0)	1			1		
12-17 (n = 130)	58 (33.3)	0.89	(0.52-1.51)		0.84	(0.45-1.60)	0.6
18-23 (n = 114)	38 (21.8)	0.55	(0.32-0.96)	0.1	0.44	(0.23-0.85)	0.02
24-59 (n = 87)	31 (17.8)	0.61	(0.34-1.10)		0.6	(0.30-1.20)	0.2

Nutritional status (n = 402)							
Marasmic (n = 110)	38 (23.0)	1			1		
Kwashiorkor (n = 162)	57 (34.6)	1.03	(0.62-1.71		1.27	(0.70-2.31)	0.8
Marasmic-kwash (n = 130)	70 (42.4)	2.2	(1.31-3.73)	0.002	2.8	(1.52-5.15)	0.001

HIV status, (n = 417)							
HIV-ve (n = 256)	91 (54.9)	1			1		
HIV+ve (n = 161)	75 (45.2)	1.58	(1.06-2.37)	0.03	1.6	(0.99-2.48)	0.06

Diarrhea, (n = 380)							
No (n = 125)	35 (21.5)	1			1		
Yes (n = 255)	128 (78.5)	2.59	(1.62-4.16)	< 0.001	2.5	(1.50-4.09)	< 0.001

Fever, (n = 379)							
No (n = 197)	92 (56.8)	1			1		
Yes (n = 182)	70 (43.2)	0.72	(0.47-1.08)	0.1	0.59	(0.37-0.93)	0.4

OR: odds ratio, n: number, -ve: negative, +ve: positive, CI: confidence Interval

**Figure 1:**
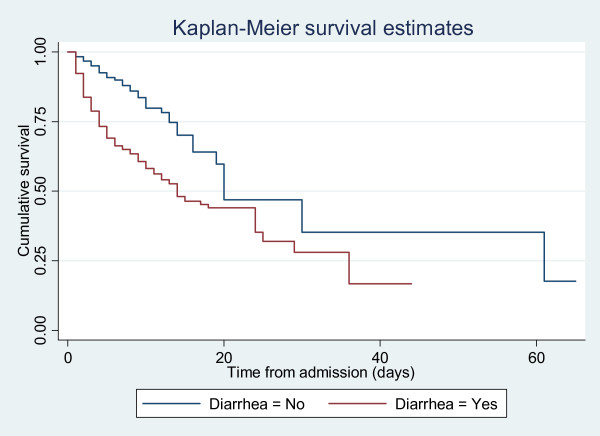
**Compares the risk of death in children with and without diarrhea**.

Figure 1 compares the risk of death in children with and without diarrhea using Kaplan-Meier survival estimates. Children with diarrhea had a significantly reduced survival rate.

## Discussion

Large number of children admitted to the stabilization centre suffered from diarrhea and HIV. The cohort also had higher prevalence of edema at admission. These factors were found to independently increase their risk of mortality during subsequent treatment at the unit. However, diarrhea was associated with the highest risk of mortality adjusted for other factors. The mortality rate observed in this study was higher than that recommended by SPHERE (less than 10%) for inpatient management of SAM[18]. It also fell short of WHO suggestions of less than 5%[9]. Similarly, the mortality rate was higher than that in other studies done in Sub-Saharan Africa; the risk of mortality in NRUs for HIV-positive children in Sub-Saharan Africa was found to be 33.6% (range: 23.6%-38.4%)[19]. The first week of inpatient stay was the most critical to the survival of children; most deaths occurred during this period. Admission fever was not a reliable predictor of mortality in these children.

The age of the children in our study was comparable to the ages of children in studies done by Bachou et al (2006) [20] and Sunguya et al (2006) [21] Children in the study by Maitland K et al (2006)[22] were older (median age of 25 months, IQR 16-46). The predominant (68.9%) form of malnutrition in our study was kwashiorkor. The study by Maitland K et al (2006)[22], however, found a relatively lower prevalence (42%) of kwashiorkor.

In a study by Maitland et al (2006)[22], the mortality rate was reduced from 30% to 19% following a stricter application of WHO therapeutic guidelines. However, the mortality rate observed in UTH had persistently been above 30% (ward audit) despite efforts to adhere to the WHO treatment recommendations. Similar high mortality rates were observed in inpatient units in Malawi[8], indicating a possibility of regional variation in case presentation and response to treatment. For a 59-bed capacity ward, the UTH inpatient unit has more children than it can accommodate year around. The congestion of the ward, with more than one child per cot, made management of the children difficult. The presence of children's caretakers and accompanying siblings has made management even more challenging. This has negatively impacted on the quality of service. The impact of such operational conditions on outcome of children treated in inpatient setups has been shown in the paper by Heikens et al, (2008)[23].

The HIV prevalence found among children in our study is higher than the 29.2% prevalence found in a recent meta-analysis[19]. Moreover, both in our study and that of Fergusson, P. & Tompkins, A., (2009)[19] it was found that HIV-positive children had a higher risk of death than HIV-negative children.

The high prevalence and observed effect of diarrhea on the outcomes of the children calls for strengthened community-level interventions, targeted towards prevention and treatment of diarrhea. Simple and effective interventions such as hand-washing, zinc supplementation, oral rehydration solutions, and water and sanitation interventions at the community level deserve a critical look[24]. This is more relevant taking into consideration the poor sanitary condition of the areas the admitted children came from[25].

Since the advent of Community-Based Therapeutic Care (CTC), the case fatality of children with SAM without complications has been reduced to less than 5% [5,26]. Accordingly more efforts need to be made in Lusaka to identify children suffering from SAM at an earlier stage by strengthening active case finding at the community level. In addition, the value of a supplementary feeding program, which is lacking in the current CTC program in Lusaka, needs to be looked into in order to prevent fast deterioration of children into severe malnutrition during peak malnutrition periods. Related to the documented high prevalence of kwashiorkor, the current practice of providing high doses of vitamin A as part of the inpatient management is questionable in the face of evidence demonstrating increased risk of mortality in children with edematous forms of malnutrition receiving high doses of vitamin A [27].

### Limitations and strength

The fact that the HIV test results presented in this paper were not confirmed by PCR, while over half of the children included in the study were less than 18 months old is a serious shortcoming of our study. Lack of key socio-demographic data might have limited our understanding of the impact such factors play on the outcome of children. Exclusion of children admitted over the weekend might have introduced a selection bias. From observation children admitted over weekends tended to be more likely to be severely ill.

This study presents findings of children with complicated SAM that receive treatment in a context where ward congestion, staff turnover and fatigue, and limited diagnostic ability exist. In addition, children came from an environment where prevalence of infectious diseases such as diarrhea and HIV are high. The operational research nature of our study, representing a real life scenario, makes it representative of most conditions in developing countries. A renewed effort to better understand the appropriate management children with complicated SAM that present with diarrhea exists, and we believe our study will add an impetus to this effort.

## Conclusion

Diarrhea is a major cause of complication in children with severe acute malnutrition admitted to the inpatient unit. Under the current standard management approach, diarrhea in children with SAM was found to increase their odds of death substantially irrespective of other factors. Given the difficulty health workers faced in treating such children- we strongly feel our findings will add value to the effort being made to improve the current management protocol for diarrhea in severely malnourished children.

## Contribution of authors

MM designed and implemented the study, and AHI proposed the study, sourced funding, and did the analysis and write-up. All authors read, critically commented, and approved the final manuscript.

## Abbreviations

CTC: Community-based Therapeutic Care; HIV: Human Immunodeficiency Virus; IQR: Inter Quartile Range; IU: International Unit; LOS: Length of Stay; MUAC: Mid-Upper Arm Circumference; NCHS: Centre for Health Statistics; OR: Odds Ration; OTP: Outpatient Therapeutic Care; PCR: Polymerase Chain Reaction; RUTF: Ready-to-Use Therapeutic Foods; UTH: University Teaching Hospital; WHO: World Health Organization; WHZ: Weight-for-Height Z-score

## Competing interests

The authors declare that they have no competing interests.
